# Bio-Inspired Genetic Algorithms with Formalized Crossover Operators for Robotic Applications

**DOI:** 10.3389/fnbot.2017.00056

**Published:** 2017-10-24

**Authors:** Jie Zhang, Man Kang, Xiaojuan Li, Geng-yang Liu

**Affiliations:** ^1^College of Information Science and Technology, Beijing University of Chemical Technology, Beijing, China; ^2^College of Information Engineering, Capital Normal University, Beijing, China

**Keywords:** genetic algorithm, formalization, crossover operator, high order logic, HOL4

## Abstract

Genetic algorithms are widely adopted to solve optimization problems in robotic applications. In such safety-critical systems, it is vitally important to formally prove the correctness when genetic algorithms are applied. This paper focuses on formal modeling of crossover operations that are one of most important operations in genetic algorithms. Specially, we for the first time formalize crossover operations with higher-order logic based on HOL4 that is easy to be deployed with its user-friendly programing environment. With correctness-guaranteed formalized crossover operations, we can safely apply them in robotic applications. We implement our technique to solve a path planning problem using a genetic algorithm with our formalized crossover operations, and the results show the effectiveness of our technique.

## Introduction

Genetic algorithms are widely adopted in robotic applications such as path planning (Hu and Yang, 2004; Taharwa et al., 2008; Achour and Chaalal, 2011; Liu et al., 2013; Sanfilippo et al., 2013; Gautam and Verma, 2014; Vicmudo et al., 2014). When genetic algorithms are applied in such safety-critical applications, it is extremely important to prove their correctness. Specially, crossover operators play a key role in searching for near-optimal solution in genetic algorithms. Therefore, it becomes an important issue for how to develop correctness-guaranteed formalized crossover operations in robotic applications (Zhou and Sun, 1999; Wang and Cao, 2002).

There have been studies to formalize crossover operations of genetic algorithms. In Uchibori and Endou (1999), completed the formalization of crossover operators. In Vidal et al. (2008), a mathematical abstraction of crossover operators is proposed to extend the applicability of formalized crossover operators in genetic algorithms. In Nawaz et al. (2013), the correctness of genetic algorithms with formalized crossover operators is verified. While the above studies lay the foundation for formalizing crossover operations of genetic algorithms, effective mechanisms and techniques are still urgently needed for developing correctness-guaranteed formalized crossover operations that can be easily deployed in genetic algorithms in practice.

In this paper, we for the first time develop correctness-guaranteed formalized crossover operations based on HOL4 (Higher-Order Logic 4) (HOL Project, 2017) that is easy to be deployed with its user-friendly programing environment. We first present a general structural model and construct the formal model of cross operators. Based on these, one-point crossover operator and multi-point crossover operator are then formalized and proved with HOL4. We conduct a case study by implementing the proposed technique in robotic applications to solve a path planning problem, in which a genetic algorithm with our formalized crossover operations has been developed, and the results show that our technique can be easily applied and effectively solve optimization problems with genetic algorithms.

The rest of paper is organized as follows. Section Manuscript Formatting presents background. Section Higher-order Logic Representation of Crossover Operators: Basic Elements describes the formal model of cross operations with HOL4. In sections Higher-order Logic Representation and Formal Verification of One-point Crossover Operators and Higher-order Logic Representation and Formal Verification of Multi-Point Crossover Operators, we formalize and prove one-point and multi-point crossover operators with HOL4, respectively. Section Discussion discusses the proposed technique. Section Evaluation evaluates the proposed work with a case study for implementing our technique to solve path planning in robotic applications. Finally, we conclude this paper in section Conclusion.

## Manuscript formatting

### Population

In order to complete the formalization of crossover operators, we must formalize the population that is the base of the evolution of genetic algorithms and the workspace of crossover operators. According to the collective property of the population, a population is defined as the abstract set and is represented as “: bool list - > bool” in HOL4. We use the symbol *D* to represent the non-empty set of a population. In addition, in order to ensure that crossover operations can be carried out in the formalized population *D* to generate new chromosomes, population *D* also needs to meet the following two properties:

- Non-unitary: There are at least two chromosomes in population *D*, and the two chromosomes are not the same.
- Closure: Offspring chromosomes generated by a crossover operator which involves two chromosomes in population *D* still belong to population *D*.

### Crossover operations

**Figure 1:**
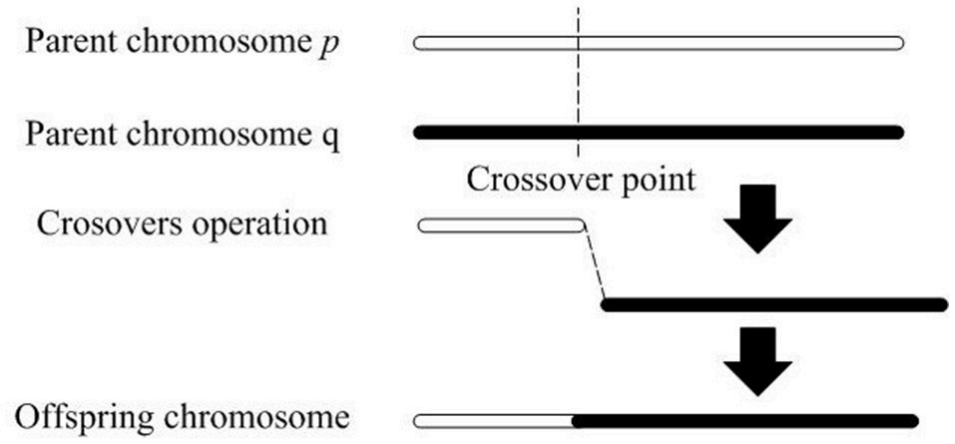
The workflow of a cross operation.

A crossover operation is defined as the behavioral process in which offspring are produced by crossover operators. A crossover operation intercepts two parent chromosomes at the crossover point, and reconnects the dissected gene segments to create a new chromosome. Figure 1 illustrate how a crossover operation works.

**Figure 2:**
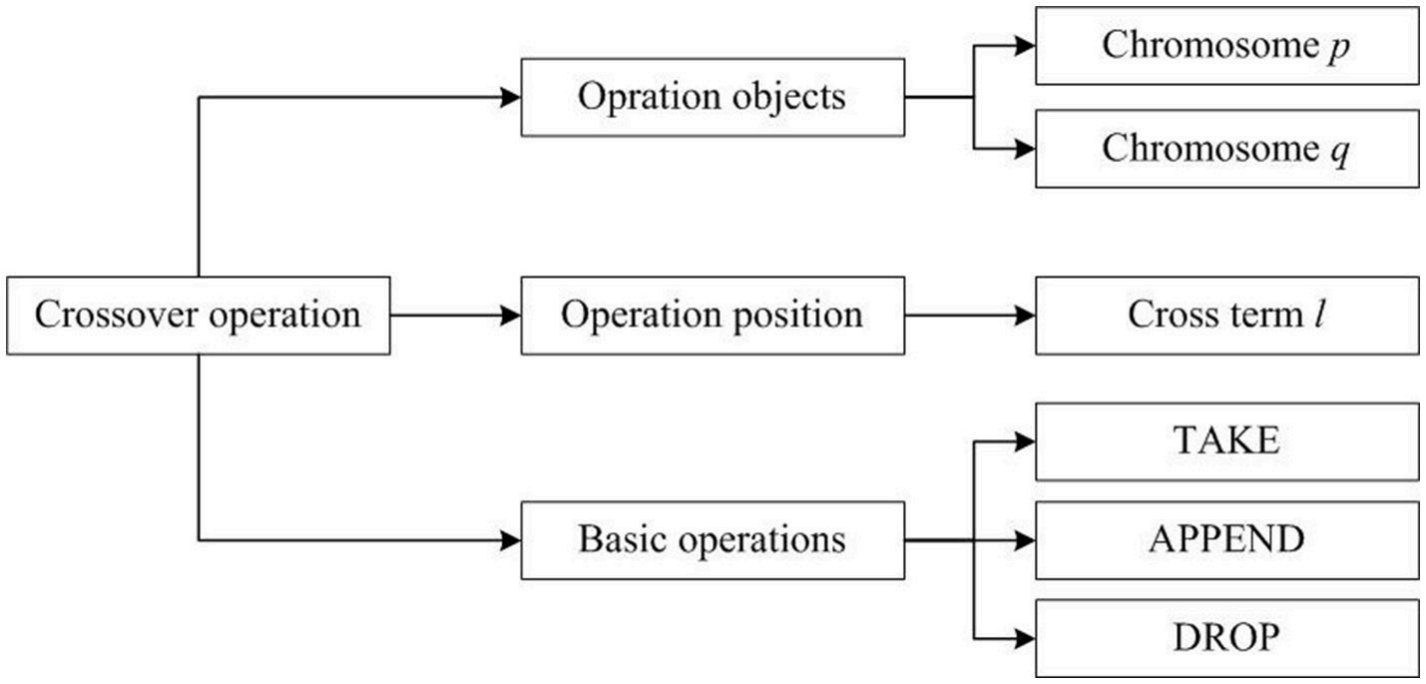
A structure model of crossover operations.

To implement the higher-order logic formalization of crossover operations, we can abstract the process shown in Figure 1. into three elements, namely, the operation object, the operation position and the basic operation. Based on this abstraction, Figure 2 shows a structural model. In Figure 2, chromosomes are individuals in population *D*; chromosome *p* and chromosome *q* as operation objects that represent the two parent chromosomes; cross-term *l* denotes the operation position which is the set of crossover points; the basic operations consisting of TAKE, DROP and APPEND are the behavior operations used to complete gene exchange.

As shown in Figure 2, the operation objects and operation positions in the general model of crossover operations constitute the basic variables of the formal model, and the basic operations in the general structural model construct the behavior of the formal model. Moreover, the basic variables and the basic behavior operations will form the formal model of the crossover operation.

## Higher-order logic representation of crossover operators: basic elements

To realize the formalization of crossover operations, the prerequisite work is to use the higher-order logic to represent the basic elements of crossover operations. Therefore, the higher-order logic representation of the three basic elements in the above model and the proofs of their related properties are presented in this section.

### Higher-order logic representation of chromosomes

Since a chromosome is an arrangement of a limited number of genes, the data structure of chromosomes in HOL4 is defined as a list; the data type of elements in the list is defined as Boolean (: bool). Then a chromosome can be represented as a Boolean list (: bool list). Correspondingly, two parent chromosomes of an operation object can be represented by *p* and *q* respectively, *p* = *p*_1_ …* p*_*n*_,*q* = *q*_1_ …* q*_*n*_,*p* ∈ *D*,*q* ∈ *D*. Here *p*_*i*_ (: bool) (*1* ≤ *i* ≤ *n*) is the gene that constitutes the chromosome *p* (: bool list);*q*_*i*_ (: bool) (*1* ≤ *i* ≤ *n*) is the gene that forms the chromosome *q* (: bool list).

### Higher-order logic representation of cross-term

The crossover position in crossover operations, called cross-term, is represented by a natural number. Thus, its data type is defined as natural number (: num) in HOL4. Since the crossover operators include one-point crossover and multi-point crossover, the number of crossover points may be one or more. Therefore, the data structure of cross-term in HOL4 is defined as natural number lists (: num list) and represented by *l* (: num list).

### Higher-order logic representation of basic operations

As described above, the data structure of both chromosomes and cross-term are defined as lists. By analyzing the list theory base in HOL4, the operation functions, namely, TAKE, DROP and APPEND, exactly match the three basic operation functions in the general model. Therefore, TAKE is used to get the first n genes of chromosome p, abbreviated as *p* ↑ *n*; DROP is used to obtain the genes after the n-th position of chromosome p, abbreviated as *q* ↓ *n*; APPEND is utilized to connect the two chromosome fragments *p*_1_ and *q*_1_ to form a new chromosome, abbreviated as *p*_1_ ++ *q*_1_.

The mathematical description of the three basic operations (TAKE, DROP and APPEND) is presented as follows.

For any *p, q* ∈ *D*, let n be the length of *p, m* the crossover point, *k* the length of *q*, where *m, n, k* ∈ *N*. The basic operations are defined as:

$$\begin{array}{l} \;p\text{ TAKE }m=\{\begin{array}{l} (p_{1},\cdots ,p_{m})\text{ if }m<n,\\ p\text{ if }m\geq n. \end{array}\\ \;p\text{ DROP }m=\{\begin{array}{l} (p_{m+1},\cdots ,p_{n})\text{ if }m<n,\\ {}[]\text{ if }m\geq n. \end{array}\\ p\text{ APPEND }q=(p_{1},\cdots ,p_{n},q_{1},\cdots ,q_{k}). \end{array}$$

Here, [] denotes an empty list.

Based on the above definitions, the higher-order logic representations of the three basic operations in HOL4 can be expressed respectively as follows:

> val TAKE =
         [] |− (!*l*. TAKE 0 *l* = []) /\
  !*n x l*. TAKE (SUC *n*) (*x*::*l*) = *x*::TAKE *n l*: thm
  > val DROP =
         [] |− (!*l*. DROP 0 *l* = *l*) /\
  !*n x l*. DROP (SUC *n*) (*x*::*l*) = DROP *n l*: thm
  > val APPEND =
         [] |− (!*l*. [] ++ *l* = *l*) /\
  !*l1 l2 h*. *h*::*l1* ++ *l2* = *h*::(*l1* ++ *l2*): thm

### Formal verification of basic operations

In HOL4 library, TAKE and DROP are used to manipulate the list, and they have two parameters, i.e., natural number and list. Function TAKE can cut the child list of list before the natural number, and function DROP can cut the child list of list after the natural number. In order to prove the properties of the formalized crossover operators, it is necessary to prove the properties of TAKE and DROP (Darmochwal and Nakamura, 1991; Kotowicz, 1993; Uchibori and Endou, 1999; Vidal et al., 2008; Nawaz et al., 2013). Since the existing properties of APPEND in HOL4 are sufficient, there is no need for more proofs. The basic properties of TAKE and DROP are classified as follows and their mathematical descriptions are given below.

Properties of TAKE: for any p, q ∈ D,m, n ∈ N

(1) ∗ []↑n=[]

(2) p↑0=[]

(3) ∗ ((p↑m)↑n)=(p↑MIN(m,n))

(4) ∗ (p↑MIN(m,n))=((p↑n)↑m)

(5) ∗ ((p↑m)↑n)=((p↑n)↑m)

(6) $$\begin{array}{l} \begin{array}{cc} \text{LENGTH} & p= \end{array}\begin{array}{cc} \text{LENGTH} & q==> \end{array}\\ {}*\;LENGTH\;(p↑n)=\text{LENGTH }(q↑n) \end{array}$$

(7) $$\begin{array}{l} \begin{array}{cc} \text{LENGTH} & \left(p↑n\right) \end{array}=\\ \text{MIN}(n,(\text{LENGTH }p)) \end{array}$$

(8) $$\begin{array}{l} ((m<=\text{LENGTH }p)/\backslash (m<=n))==>\\ {}*\;(m<=\text{LENGTH }(p↑n)) \end{array}$$

(9) $$\begin{array}{l} (m<=\begin{array}{cc} \text{LENGTH} & p \end{array})==>\\ {}*\;(m=\begin{array}{cc} \text{LENGTH} & \left(p↑m\right) \end{array}) \end{array}$$

(10) $$\begin{array}{l} (\begin{array}{cc} \text{LENGTH} & p \end{array}<=n)==>\\ ((p++q)↑n=p++\\ {}*\;(q↑(n-\begin{array}{cc} \text{LENGTH} & p \end{array}))) \end{array}$$

(11) $$\begin{array}{l} \begin{array}{cc} \text{LENGTH} & \left(p↑n\right) \end{array}=\\ \text{if}(n<=\begin{array}{cc} \text{LENGTH} & p \end{array})\\ \text{then }n\\ \text{else }(\text{LENGTH }p) \end{array}$$

(12) $$\begin{array}{cc} (\text{LENGTH} & p \end{array})<=n==>p↑n=p$$

(13) $$\begin{array}{l} (n<=\begin{array}{cc} \text{LENGTH} & p \end{array})==>\\ \left((p++q)↑n=p↑n\right) \end{array}$$

(14) $$\begin{array}{l} (\begin{array}{cc} \text{LENGTH} & p \end{array}<n)==>\\ ((p++q)↑n=p++\\ \left(q↑(n-\begin{array}{cc} \text{LENGTH} & p \end{array}))\right) \end{array}$$

(15) $$\begin{array}{l} (\begin{array}{cc} \text{LENGTH} & p \end{array}<n)==>\\ ((p++q)↑n=p++\\ \left(q↑(n-\begin{array}{cc} \text{LENGTH} & p \end{array}))\right) \end{array}$$

(16) $$\begin{array}{l} (m<=\begin{array}{cc} \text{LENGTH} & p)\wedge (n<=m)==> \end{array}\\ \left(((p↑m)↑n)=p↑n\right) \end{array}$$

Properties of DROP: for any *p, q* ∈ *D, m, n* ∈ *N*

(17) ∗ []↓n=[]

(18) p↓0=p

(19) p↓0=q==>p=q

(20) ∗ ((p↓m)↓n)=(p↓(m+n))

(21) ∗ (p↓(m+n))=((p↓n)↓m)

(22) ∗ ((p↓m)↓n)=((p↓n)↓m)

(23) $$\begin{array}{l} \begin{array}{cc} \text{LENGTH} & p= \end{array}\begin{array}{cc} \text{LENGTH} & q==> \end{array}\\ \begin{array}{cc} {}*\;LENGTH & (p↓n \end{array})=\begin{array}{cc} \text{LENGTH} & (q↓n \end{array}) \end{array}$$

(24) $$\begin{array}{cc} \text{LENGTH} & (p↓n)=\begin{array}{cc} (\text{LENGTH} & p)-n \end{array} \end{array}$$

(25) $$\begin{array}{cc} (\text{LENGTH} & p \end{array})<=n==>p↓n=[]$$

(26) $$\begin{array}{l} (n<=\begin{array}{cc} \text{LENGTH} & p \end{array})==>\\ \left((p++q)↓n=(p↓n)++q\right) \end{array}$$

(27) $$\begin{array}{l} (\begin{array}{cc} \text{LENGTH} & p \end{array}<=n)==>\\ \left((p++q)↓n=q↓(n-\begin{array}{cc} \text{LENGTH} & p \end{array})\right) \end{array}$$

(28) $$\begin{array}{l} ((n+m)<=\begin{array}{cc} \text{LENGTH} & p)==> \end{array}\\ \left(((p↓m)↓n)=p↓(n+m)\right) \end{array}$$

The relation between TAKE and DROP: for any *p*, *q* ∈ *D,m,n* ∈ *N*

(29) ∗ (p↑n)↓n=[]

(30) ∗ (p↓n)↑m=(p↑(m+n))↓n

(31) ∗ (p↑n)↓m=(p↓m)↑(n−m)

In the above equations, the properties with ^*^ are required to be proved in this paper, while these properties without ^*^ have existed in HOL4 and need not be proved.

### Formal modeling and implementation of crossover operations with HOL4

**Figure 3:**
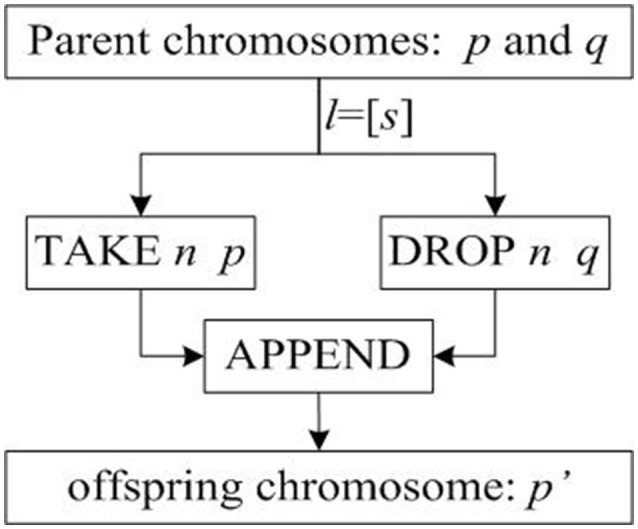
Basic flow chart of crossover operations.

As mentioned above, crossover operations are the process of generating offspring. In order to establish a formal model of crossover operation, we first construct the basic implementation flow of generating offspring based on the general structural model of crossover operation, as shown in Figure 3.

In Figure 3, *p* and *q* are two parent chromosomes; *l* is the cross-term that represents the crossover position; l = [s] indicates that there is only one crossover point s; chromosome p' is the offspring chromosome generated.

The basic implementation of crossover operation in Figure 3 can only be used for one-point crossover operator. In order to apply the formalized crossover operation to other crossover operators, the crossover process is improved according to the characteristics of multi-point crossover operators.

In general, the process of multi-point crossover can be regarded as the repetition of one-point crossover. Therefore, when the number of crossover points are *n* (*n* > 1) in cross-term l, the operation objects of TAKE and DROP are the offspring chromosomes generated by *n-*1 rounds of crossover.

**Figure 4:**
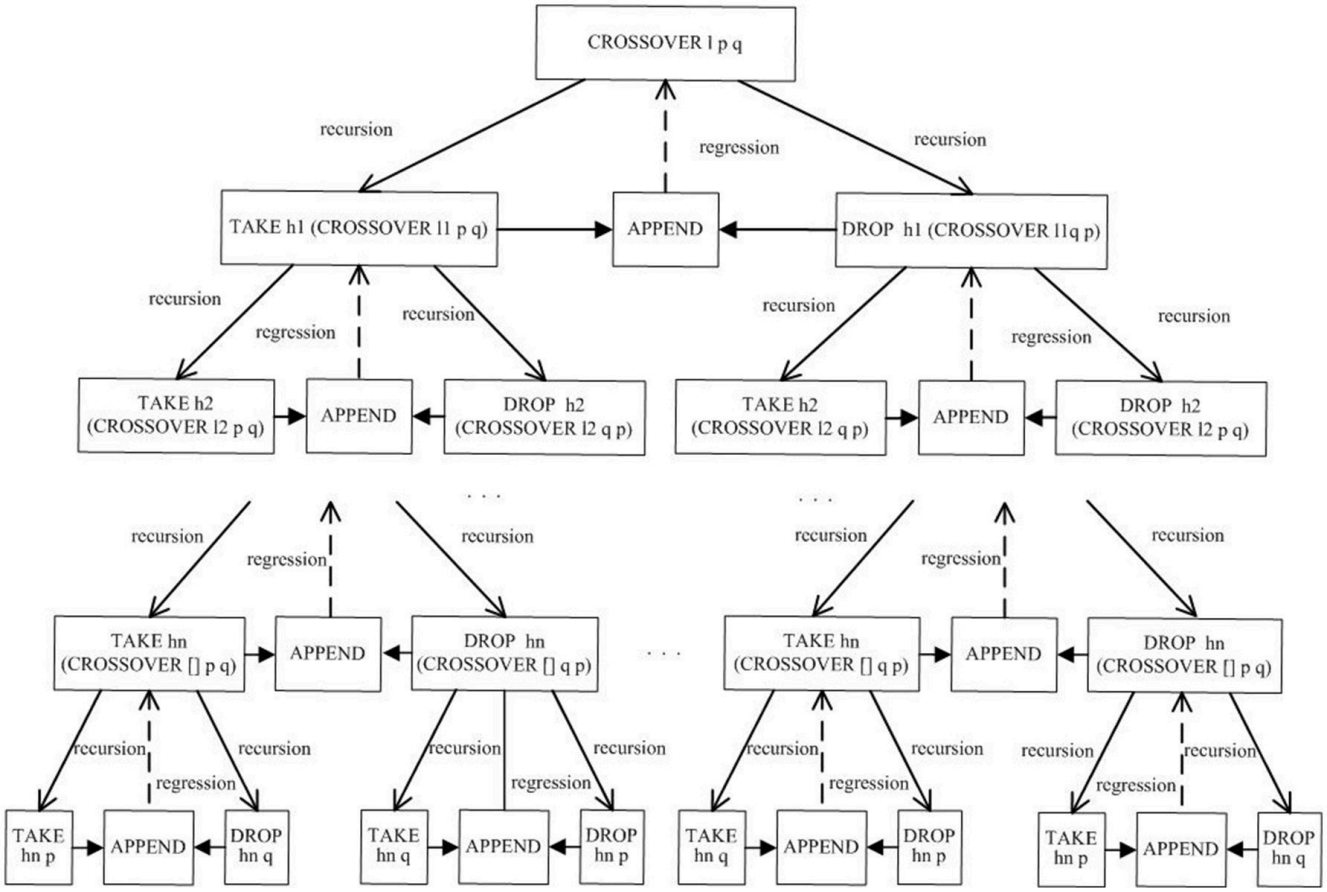
Implementation flow of crossover operations.

Let CROSSOVER represent a crossover operation. CROSSOVER crosses the chromosomes *p* and *q* in turns according to the crossover points in cross-term *l*. According to the features of the functional language, recursive methods can be used to achieve the repeated process between one-point crossover and multi-point crossover. Figure 4 shows the implementation process of crossover operations.

As shown in Figure 4, CROSSOVER *l p q* is the offspring chromosome generated by the crossover operation with two parent chromosomes *p* and *q*. Similarly, CROSSOVER *l q p* is another offspring chromosome generated by the crossover operation with two parent chromosomes *q* and *p*. To complete the gene exchange, a crossover operation uses two basic operations, namely, TAKE and DROP. The operation object of TAKE and DROP also contains the crossover operation itself, so the whole process contains two recursive lines. Because the two recursive lines are parallel, the method employed is called double recursion. According to the execution diagram of the double recursion, it can be observed that the recursion procedure is to reduce the size of the cross-term, while the regression process is to exchange genes at each crossover point in turn.

In Figure 4, the implementation procedure of the crossover operation can be viewed as a binary tree where the number of crossover points corresponds to the height of the binary tree. For the special case in which there is only one crossover point, the height of the full binary tree is one. Therefore, the crossover operation with the double recursion method, which can be used to construct one-point crossover and multi-point crossover, possesses generality. Moreover, the implementation process of this crossover operation can also be used to form other crossover operators such as uniform crossover operators and partially matched crossover operators.

According to the implementation process of the crossover operation, the mathematical description of the crossover operation is given as follows:

$$\text{CROSSOVER }l\text{ p q}=\;\{\begin{array}{ll} p & \text{if }l=[],\\ \left((\text{CROSSOVER }t\;p\;q)↑h\right) & \text{if }l=h::t.\\ ++((\text{CROSSOVER }t\;q\;p)↓h) & \end{array}$$

Based on the above mathematical description, the higher-order logic implementation of the crossover operation in HOL4 is given as follows:

> val CROSSOVER_def =
         [] |− (!*p q*. CROSSOVER [] *p q* = *p*) /\
         !*h t p q*.
         CROSSOVER (*h*::*t*) *p q* =
         TAKE *h* (CROSSOVER *t p q*) ++
      DROP *h* (CROSSOVER *t q p*): thm

The higher-order logic description of the crossover operation is an important preliminary work for formalizing crossover operators. We further describe the one-point crossover operator and multi-point crossover operator using higher-order logic in HOL4 and complete the proofs of their relevant properties next.

## Higher-order logic representation and formal verification of one-point crossover operators

One-point crossover operator selects two chromosomes in population D as two parent chromosomes and one random crossover point, and then exchanges the chromosome segments at the crossover point to obtain two new offspring chromosomes.

Two parent chromosomes in population D are defined as follows:

$$\begin{array}{l} p=p_{1},p_{2},\ldots ,p_{n}\\ q=q_{1},q_{2},\ldots ,q_{n} \end{array}$$

*p* and *q* represent the two parent chromosomes; *p*_*i*_ (1 ≤ *i* ≤ n) and *q*_*i*_ (1 ≤ *i* ≤ n) express the genes that make up the chromosomes.

Choose an random intersection *i*(1 ≤ *i* ≤ n), then generate two new offspring:

$$\begin{array}{l} p^{\prime }=p_{1},\ldots ,p_{i},q_{i+1},\ldots ,q_{\text{n}}\\ q^{\prime }=q_{1},\ldots ,q_{i},p_{i+1},\ldots ,p_{\text{n}} \end{array}$$

*p*′ and *q*′ denote the two offspring; *p*_*i*_ (1 ≤ *i* ≤ n) and *q*_*i*_ (1 ≤ *i* ≤ n) express the genes that make up the chromosomes.

### Formalization of one-point crossover operator in HOL4

From the definition of the one-point crossover operator, it is known that the one-point crossover operator generates two chromosomes at the same time, while the crossover operation can only produce one offspring chromosome every time. Thus, the implementation of the one-point crossover operator needs two crossover operations. Since the two offspring are generated at the same time and their relation is parallel, two-tuples are used to indicate the relation between two offspring generated in the mathematical description of the one-point crossover operator as follows:

$$\begin{array}{l} \begin{array}{c} \bar{⊙}\;n\;p\;q \end{array}=\begin{array}{c} (\text{CROSSOVER}\;[n]\;p\;q, \end{array}\\ \begin{array}{c} \text{ CROSSOVER}\;[n]\;q\;p \end{array}). \end{array}$$

Symbol $\bar{⊙}$ represents an one-point crossover operator; CROSSOVER denotes the crossover operation; *p* and *q* are two parent chromosomes in population *D*; [*n*] is the crossover term with one crossover point *n*.

Based on the above mathematical descriptions, the higher-order logic description of the one-point crossover operator in HOL4 is as follows:

> val ONEPOINT_CROSSOVER_def =
         [] |− !*n p q*.
         ONEPOINT_CROSSOVER *n* (*p,q*) =
  (CROSSOVER [*n*] *p q*, CROSSOVER [*n*] *q p*): thm

### Verification of one-point crossover operator

In order to ensure the correctness of one-point crossover operator, we prove the four basic properties of the one-point crossover operator in HOL4.

Theorem 1: Given any *p, q* ∈ *D* and a random crossover point *n*, if LENGTH*p* = LENGTH*q*, then LENGTH (CROSSOVER [*n*] *p q*) = LENGTH *p*. The higher-order logic description is as follows:

> val OCROSSOVER_LENGTH =
         [] |− !*n p q*.
         *p* IN *D* /\*q* IN *D* /\
         (LENGTH *p* = LENGTH *q*) ==>
         (LENGTH (CROSSOVER [*n*] *p q*) =
         LENGTH *p*): thm

Theorem 1 ensures that the one-point crossover does not change the length of chromosome.

Theorem 2: Given any *p, q* ∈ *D*, if LENGTH *p* = LENGTH *q*, 0 is the crossover point, then CROSSOVER [0] *p q* = *q*. The higher-order logic description is as follows:

> val OCROSSOVER_ZERO =
         [] |− !*p q*.
         *p* IN *D* /\*q* IN *D*
         /\(LENGTH *p* = LENGTH *q*) ==>
         (CROSSOVER [0] *p q* = *q*): thm

Theorem 2 shows that when crossover point is 0, the offspring generated by one-point crossover operator are the same as parent chromosomes, but the order is exchanged, that is, the first offspring is the second parent and the second child is the first parent.

Theorem 3: Given any *p, q* ∈ *D*, if LENGTH *p* = LENGTH *q*, *n* is the crossover point andLENGTH *p* < *n*, then CROSSOVER [*n*] *p q* = *p*. The higher-order logic description is as follows:

> val OCROSSOVER_TOO_LONG =
         [] |− !*n p q*.
         *p* IN *D* /\*q* IN *D* /\
         (LENGTH *p* = LENGTH *q*) /\
         LENGTH *p* < *n* ==>
         (CROSSOVER [*n*] *p q* = *p*): thm

Theorem 3 guarantees that if the position of the crossing point is larger than the length of chromosome, then the offspring produced by the one-point crossover operator are the same as the parent chromosomes.

Theorem 4: Given any *p, q* ∈ *D*, if LENGTH *p* = LENGTH *q*, *n* is the crossover point and LENGTH *p* = *n*, then CROSSOVER [*n*] *p q* = *p*. The higher-order logic description is as follows:

> val OCROSSOVER_EQ =
            [] |− !*n p q*.
            *p* IN *D* /\*q* IN *D* /\
         (LENGTH *p* = LENGTH *q*) /\
         (*n* = LENGTH *p*) ==>
         (CROSSOVER [*n*] *p q* = *p*): thm

Theorem 4 holds the property that if the position of the intersection is equal to the length of chromosome, the offspring generated by the one-point crossover operator are the same as the parents.

As mentioned above, Theorems 1-4 mainly reflect the relation between the positions of crossover points and the results produced by the one-point crossover operator. In addition, the formalization of the one-point crossover operator provides a good foundation for our analysis and design of formalization of the multi-point crossover operator.

## Higher-order logic representation and formal verification of multi-point crossover operators

With a multi-point crossover operator, two chromosomes in population *D* are selected as two parent chromosomes, and with n crossover points, we exchange the chromosome segments to eventually obtain two new offspring chromosomes.

Two parent chromosomes in population *D* are:

$$\begin{array}{l} p=p_{1},p_{2},\ldots ,p_{\text{n}}\\ q=q_{1},q_{2},\ldots ,q_{\text{n}} \end{array}$$

*p* and *q* represent the two parent chromosomes; *p*_*i*_ (1 ≤ *i* ≤ n) and *q*_*i*_ (1 ≤ *i* ≤ n) represent the genes that make up the chromosomes.

By randomly selecting *n* crossover points: *i, j, k*,…., the offspring produced can be represented as follows:

$$\begin{array}{l} p^{\prime }=p_{1},\ldots ,p_{i},q_{i+1},\ldots ,q_{j},p_{j+1},\ldots ,p_{k},q_{k+1}\ldots \\ q^{\prime }=q_{1},\ldots ,q_{i},p_{i+1},\ldots ,p_{j},q_{j+1},\ldots ,q_{k},p_{k+1}\ldots \end{array}$$

Here, *p*′ and *q*′ denote the two offspring; *p*_*i*_ (1 ≤ *i* ≤ n).

### Formalization of multi-point crossover operator in HOL4

From the definition of the multi-point crossover operator, we can see that the multi-point crossover operator is similar to the one-point crossover operator. In both cases, progeny is generated in parallel. The difference is the number of crossover points. Therefore, the creation of multi-point crossover also needs two crossover operations. However, the cross-term denoted by *l* is an arrangement of multiple crossover points rather than only one point. When describing the multi-point crossover with mathematical methods, we still use two-tuples to represent the parallel relation between two offspring.

Therefore, the mathematical description of the multi-point crossover operator obtained is as follows:

$$\bar{⊗}\;l\;(p,q)=(\text{CROSSOVER }l\;p\;q\;,\;CROSSOVER\;l\;q\;p).$$

Symbol $\bar{⊗}$ represents multi-point crossover operator; CROSSOVER denotes crossover operation; *p* and *q* are two parent chromosomes in population *D*; *l* is the cross-term with multiple crossover points.

Based on the above mathematical description, a multi-point crossover operator in HOL4 can be denoted as follows:

> val MULTIPOINT_CROSSOVER_def =
         [] |− !*l p q*.
         MULTIPOINT_CROSSOVER *l* (*p*,*q*) =
         (CROSSOVER *l p q*, CROSSOVER *l q p*): thm

### Verification of multi-point crossover operator

To ensure that the higher-order logic representation of a multi-point crossover operator is correct, its properties must be verified. In the following, Theorems 6 and 7 describe the relation between the crossover point and offspring generated by the multi-point crossover operator; Theorems 8-9 and Theorems 14-16 mainly show that the results produced by the multi-point crossover operator are independent of the arrangement of cross-term; Theorem 10 guarantees that the elimination of two identical elements in a cross-term does not affect the results obtained by the multi-point crossover operator.

Theorem 5: Given any *p, q* ∈ *D*, any cross-term *l*, if LENGTH *p* = LENGTH *q*, then LENGTH (CROSSOVER *l p q*) = LENGTH *p*. The higher-order logic description is as follows:

> val XCROSSOVER_LENGTH =
            []|− !*l p q*.
            *p* IN *D* /\*q* IN *D* /\
         (LENGTH *p* = LENGTH *q*) ==>
         (LENGTH (CROSSOVER *l p q*) = LENGTH *p*): thm

Theorem 5 ensures that the length of new chromosomes generated by the multi-point crossover is equal to the length of two parent chromosomes *p* and *q*.

Theorem 6: Given any *p, q* ∈ *D*, any cross-term *l*, if LENGTH *p* = LENGTH *q*, then CROSSOVER (0::*l*) *p q* = CROSSOVER *l q p*. The higher-order logic description is as follows:

> val XCROSSOVER_ZERO_APPEND =
            [] |− !*p q l*.
            *p* IN *D* /\*q* IN *D* ==>
         (CROSSOVER (0::*l*) *p q* = CROSSOVER *l q p*): thm

Theorem 6 shows that in the case of the same chromosomes *p* and *q*, adding a crossover point 0 at the beginning of the cross-term *l* does not change the progeny generated by the multi-point crossover.

Theorem 7: Given any *p, q* ∈ *D*, any cross-term *l*, if LENGTH *p* = LENGTH *q* and LENGTH *p* ≤ *n*, then CROSSOVER (*n*::*l*) *p q* = CROSSOVER *l p q*. The higher-order logic description is as follows:

> val XCROSSOVER_TOO_LENGTH =
            [] |− !*p q n l*.
            *p* IN *D* /\*q* IN *D* /\
         (LENGTH *p* = LENGTH *q*) /\LENGTH *p* < = *n* ==>
         (CROSSOVER (*n*::*l*) *p q* = CROSSOVER *l p q*): thm

Theorem 7 guarantees that in the case of the same parent chromosomes *p* and *q*, when *n* is not less than the length of chromosome, adding a crossover point *n* at the beginning of the cross-term *l* does not change the progeny generated by multi-point crossover.

Theorem 8: Given any *p, q* ∈ *D*, any cross-term *l, l1, l2*, if LENGTH *p* = *cc*LENGTH *q* and CROSSOVER *l*1 *p q* = CROSSOVER *l*2 *p q*, then CROSSOVER (*l*++*l*1) *p q* = CROSSOVER (*l*++*l*2) *p q*. The higher-order logic description is as follows:

> val XCROSSOVER_EQ =
            []|− !*l1 l2*.
            (!*p q*.*p* IN *D* /\*q* IN *D* /\
            (LENGTH *p* = LENGTH *q*) /\
         (CROSSOVER *l1 p q* = CROSSOVER *l2 p q*)) ==>
            !*p q l*. CROSSOVER (*l* ++ *l1*) *p q* =
         CROSSOVER (*l* ++ *l2*) *p q*: thm

Theorem 8 shows that in the case of the same parent chromosomes *p* and *q*, if the offspring generated by the multi-point crossover with cross-term *l1* are equal to the offspring produced by the multi-point crossover with cross-term *l2*, adding cross-term *l* at the beginning of the cross-term *l1* and *l2* respectively does not change the equivalency of the progeny generated in the same way.

Theorem 9: Given any *p, q* ∈ *D*, any cross-term *l*, any *m, n* ∈ *N*, if LENGTH *p* = LENGTH *q*, then CROSSOVER (*n*::(*m*::*l*)) *p q* = CROSSOVER (*m*::(*n*::*l*)) *p q*. The higher-order logic description is as follows:

> val XCROSSOVER_SWAP =
            [] |− !*p q l m n*.
            *p* IN *D* /\*q* IN *D* /\
         (LENGTH *p* = LENGTH *q*) ==>
            (CROSSOVER (*n*::*m*::*l*) *p q* =
            CROSSOVER (*m*::*n*::*l*) *p q*): thm

Theorem 9 holds the property that in the case of the same parent chromosomes *p* and *q*, two crossover points with different order are added respectively at the beginning of cross-term *l*, then the progeny produced by the multi-point crossover with the new two cross-terms respectively are the same.

Theorem 10: Given any *p, q* ∈ *D*, any cross-term *l*, any *n* ∈ *N*, if LENGTH *p* = LENGTH *q*, then CROSSOVER (*n*::(*n*::*l*)) *p q* = CROSSOVER *l p q*. The higher-order logic description is as follows:

> val XCROSSOVER_SAME =
            [] |− !*p q l n*.
            *p* IN *D* /\*q* IN *D* /\
            (LENGTH *p* = LENGTH *q*) ==>
         (CROSSOVER (*n*::*n*::*l*) *p q* = CROSSOVER *l p q*): thm

Theorem 10 guarantees that in the case of the same parent chromosomes *p* and *q*, if we add two identical crossover points at the beginning of the cross-term *l* to obtain a new cross-term, the offspring generated by multi-point crossover operator with the new cross-term are the equal to those generated with cross-term *l*. In other words, the elimination of two identical elements in cross-term does not affect the results generated by multi-point crossover.

From Theorems 8–10, we can see that the results produced by the multi-point crossover operator used in genetic algorithms are related to the position of crossover points, and independent of the order of crossover points.

To demonstrate that the results generated by the multi-point crossover are not affected by the order of cross-term, the concept of strictly increasing list is used to verify the properties of the crossover operator. Strictly increasing list means that the elements in the list are in ascending order, and two identical elements are eliminated in the list. If cross-term *l'* is the strictly increasing list of cross-term *l*, the property that progeny generated by the multi-point crossover operator are not affected by the order of cross-term, can be expressed as: CROSSOVER *l p q* = CROSSOVER *l*′ *p q*.

In order to get the strictly increasing list of any list in HOL4, it is necessary to define two predicates INSERT_PL and CANON_PL. Given a strictly increasing list *l* and a natural number *n*, when *n* is not in list *1*, predicate INSERT_PL can produce a new strictly increasing list with element *n*; otherwise it gets a new list that has the eliminated element *n*. The function of predicate CANON_PL is to get the strictly increasing list *l'* of any given list *l*.

The higher-order logic presentations of predicate INSERT_PL and predicate CANON_PL are expressed as follows:

> val INSERT_PL =
         [] |− (!*n*. INSERT_PL *n* [] = [*n*]) /\
            !*n h t*.
         INSERT_PL *n* (*h*::*t*) =
         if *n* < *h* then *n*::*h*::*t* else if *n* = *h*
         then *t* else *h*::INSERT_PL *n t*: thm
  > val CANON_PL =
         [] |− (CANON_PL [] = []) /\
            !*h t*. CANON_PL (*h*::*t*) =
         INSERT_PL *h* (CANON_PL *t*): thm

To ensure that the definitions of the two predicates are correct, we need to prove the properties of the strictly increasing list. The property of the strictly increasing list can be described as “any element in a strictly increasing list is smaller than the next element”. Predicate INCREASE_PRO is used to represent this property in HOL4, and its higher-order logic description is as follows:

> val INCREASE_PRO =
         [] |- (INCREASE_PRO [] <=> T) /\
            !*t1 h1*. INCREASE_PRO (*h1*::*t1*) <=>
         case *t1* of [] = > T |*h2*::*t2* = >
       *h1* < *h2* /\INCREASE_PRO (*h2*::*t2*): thm

In addition, it is also required to prove the following properties of the strictly increasing list:

Theorem 11: Given any cross-term *l*, any *m, n* ∈ *N*, if INCREASE_PRO(*n*::*l*) and *m* < *n*, then INCREASE_PRO(*m*::*l*). The higher-order logic description is as follows:

> val LIST_INCREASE_ONE =
         [] |− !*l m n*. INCREASE_PRO (*n*::*l*) /\
       *m* < *n* ==> INCREASE_PRO (*m*::*l*): thm

Theorem 11 shows that if adding a natural number *n* at the beginning of list *l* possesses the strictly increasing property, then adding a natural number *m* that is smaller than *n* at the beginning of list *l* can still get a new strictly increasing list.

Theorem 12: Given any cross-term *l*, any *n* ∈ *N*, if INCREASE_PRO *l*, then INSERT_PL *n l* still meets INCREASE_PRO(INSERT_PL *n l*). The higher-order logic description is as follows:

> val LIST_INCREASE_INSERT =
         [] |- !*l n*. INCREASE_PRO *l* ==>
       INCREASE_PRO (INSERT_PL *n l*): thm

Theorem 12 ensures that if *l* is a strictly increasing list and a natural number *n* is inserted into list *1* by predicate INSERT_PL, then the new list obtained is still a strictly increasing list.

In order to prove the theorem 13, we need to give the following lemma:

Lemma 1: Given any cross-term *l*, any *m, n* ∈ *N*, if INCREASE_PRO(*m*::*n*::*l*), then *m* < *n* and INCREASE_PRO(*n*::*l*). The higher-order logic description is as follows:

> val LIST_INCREASE_IMP =
         [] |− !*l m n*. INCREASE_PRO (*m*::*n*::*l*) ==>
         *m* < *n* /\INCREASE_PRO (*n*::*l*): thm

Lemma 1 shows that if (*m*::*n*::*l*) is a strictly increasing list, then (*n*::*l*) is a strictly increasing list and *m* < *n*.

Theorem 13: Given any cross-term *l*, if INCREASE_PRO *l*, then CANON_PL *l* = *l*. The higher-order logic description is as follows:

> val LIST_INCREASE_CANON = [] |−
  !*l*. INCREASE_PRO *l* ==> (CANON_PL *l* = *l*): thm

Theorem 13 illustrates that if the arrangement of a list is strictly incremental, then the list is a strictly increasing list.

The proofs of Theorems 11–13 ensure the correctness of the strictly increasing list defined in HOL4. By using the definition of strictly increasing list, the following properties of the multi-point crossover operator can be further proved.

Theorem 14: Given any *p, q* ∈ *D*, any cross-term *l*. *l*′ is the strictly increasing list of *l*, if LENGTH *p* = LENGTH *q*, then CROSSOVER *l p q* = CROSSOVER*l*′ *p q*. The higher-order logic description is as follows:

> val CANON_XCROSSOVER_EQ =
         [] |− !*p q l n*.
         *p* IN *D* /\*q* IN *D* /\
         (LENGTH *p* = LENGTH *q*) ==>
         (CROSSOVER (CANON *l*) *p q* =
         CROSSOVER *l p q*): thm

Theorem 14 guarantees that in the case of the same parent chromosomes *p* and *q*, the progeny generated by multi-point crossover with cross-term *l* are equal to the ones produced in the same way with the strictly increasing list of *l*. It is also more straightforward to illustrate that the results of the multi-point crossover are independent of the order of the elements in the cross-term.

Theorem 15: Given any *p, q* ∈ *D*, any cross-term *l*. *l*′ is the strictly increasing list of *l*, any *n* ∈ *N*. if LENGTH *p* = LENGTH *q*, then CROSSOVER (INSERT_PL *n l*′) *p q* = CROSSOVER (*n*::*l*) *p q*. The higher-order logic description is as follows:

> val LINCREASE_XCROSSOVER_N =
         [] |− !*p q l n*.
         *p* IN *D* /\*q* IN *D* /\
         (LENGTH *p* = LENGTH *q*) ==>
         (CROSSOVER (INSERT_PL *n l*) *p q* =
         CROSSOVER (*n*::*l*) *p q*): thm

Theorem 15 shows that in the case of the same parent chromosomes *p* and *q*, if the crossover point *n* is inserted into cross-term *l* and *l'* respectively, where *l'* is the strictly increasing list of *l*, then the results obtained by the multi-point crossover under these two new cross-terms are the same.

Theorem 16: Given any *p, q* ∈ *D*, any cross-term *l1, l2*, if LENGTH *p* = LENGTH *q* and (CANON_PL *l*1) = (CANON_PL *l*2), then CROSSOVER *l*1 *p q* = CROSSOVER *l*2 *p q*. The higher-order logic description is as follows:

> val CANON_XCROSSOVER_DEQ =
  [] |− !*p q l1 l2*.
  *p* IN *D* /\*q* IN *D* /\
  (LENGTH *p* = LENGTH *q*) /\
  (CANON_PL *l1* = CANON_PL *l2*) ==>
  (CROSSOVER *l1 p q* = CROSSOVER *l2 p q*): thm

Theorem 16 ensures that in the case of the same parent chromosomes *p* and *q*, if the strictly increasing list of different cross-terms are the same, then the offspring respectively produced by the multi-point crossover under the different cross-terms are the same.

## Discussion

As shown above, one-point and multi-point crossover operators are formalized and verified. The proposed technique is general and can applied in formalizing and verifying other genetic operators in genetic algorithms such as mutation. Mutation is another genetic operator that can preserve genetic diversity in such a way local minima caused by similar populations of chromosomes can be avoided. With mutation, one or more gene values in a chromosome can be changed from its initial state so a better solution may be achieved. To implement mutation, a common method is to generate a random variable for each bit in a chromosome sequence that is used to determine whether or not a particular bit will be amended. To realize the formalization and verification of mutation operations, first, we need to use the higher-order logic to represent basic elements, and then we can construct the formal modeling and perform verification with HOL4. This will be investigated in our future work.

## Evaluation

### Experimental setup

We conduct the experiment in Windows 7 with the specific tool HOL4, and the programing language is ML. In the experiment, robot path planning based on GA is described by ML, and the cross operation in algorithm is described by multi-point crossover mentioned in this paper. We proved the effectiveness of our formal model of crossover by running the algorithm in HOL4 successfully. In addition, we do not need to specify the inputs because of the advantage of theorem proving, and HOL4 will exhaust all the cases space, i.e., covering all inputs. The final output will show that robot can avoid collisions in any input situation.

### Case study on robotics

Genetic Algorithms have many advantages compared with traditional optimization methods. In this section, we present a case study on robotics, in which a genetic algorithm with the two-point crossover operator implemented by HOL4 is applied in robot path planning. We give the specific formal description and collision free verification.

**Figure 5:**
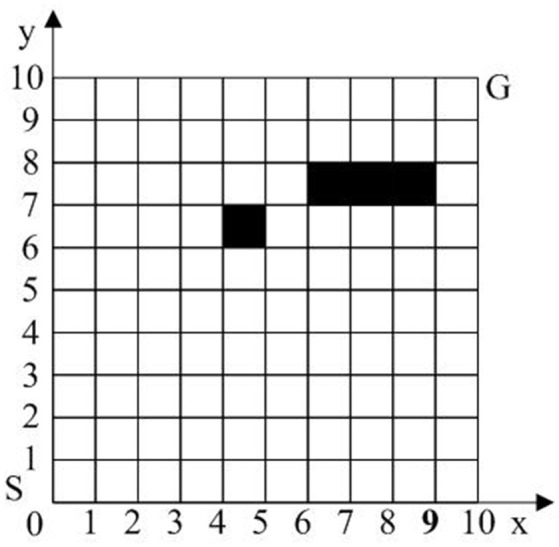
Schematic diagram of the robot motion space.

The workspace of the robot is a 2-D environment. We assume that the location and size of obstacles are known, and the obstacles will stay the same during the movement of robot. The robot working space is modeled with grids following Cartesian coordinates. As shown in Figure 5, the lower left corner of the grid array is the coordinate origin, the right direction of the horizontal axis is the forward direction of x axis, and the up direction of the vertical axis is the forward direction of y axis. Each grid interval corresponds to a unit length on the coordinate axis, and any grid can be uniquely identified by (*x, y*). The length and width of a grid is defined as 10 units of distance, S represents the starting point of the robot, G represents the target point of the robot, and black grids are used to represent obstacles.

The moving path of the robot is represented by the chromosome. We defined the chromosome through a real list, where the subscript of the list represents the value of the coordinate *x*, and the gene is the value of the list, which represents the value of the coordinate *y*. The population is composed of a certain number of chromosomes, and its size is 20. In order to guarantee the global optimality of the genetic algorithm, the initial population is randomly generated.

The fitness function directly affects the computation efficiency and time of the genetic algorithm. In the path planning of the robot, the design of the fitness function needs to consider the length of the path and collision avoidance. Therefore, the fitness function is set to the sum of the path length and the obstacles' coordinates. It is represented as:

$$L=L1+L2=\sum_{i=1}^{N}\sqrt{{\left(y_{i+1}-y_{i}\right)}^{2}+1}+A\sum_{j=1}^{m}\left(x_{j}+y_{j}\right)$$

Here, *L1* represents the distance between two adjacent coordinate points, and *L2* represents the obstacle coordinates. When *i* = *xj* and *yi* = *yj, A* is 100; otherwise *A* is 0. *N* = 10 indicates that the space coordinate has 10 unit lengths,*m* represents the number of obstacles. In this way, the fitness function is very large when there are obstacles in the path. In order to simplify the calculation in HOL4, we modified the function as follows:

$$L^{\prime }=\sum_{i=1}^{N}{\left(y_{i+1}-y_{i}\right)}^{2}+10+A\sum_{j=1}^{m}\left(x_{j}+y_{j}\right)$$

In this function, *L1* represents the sum of squares of the distance between two points. Although we modified the calculated way of *L1*, it does not affect the comparative relationship between the length of the path of two chromosomes. Thus, the new fitness function can also be used to evaluate the optimal path in the path planning. According to the property of the shortest path, we can see that the lower the fitness value of the chromosome is, the better the path will become.

In this genetic algorithm, we use three basic genetic operators: selection, crossover, and mutation. In the selection operator, we use ranking selection, by which each individual in the population is ranked from low to high according to the fitness, the selected probability of the forward 80% individuals in the population is 6.25%, and the remaining 20% is 0. In the crossover operator, we use the two-point crossover operator, the crossover probability is 0.9, and the two intersections are generated randomly. In the mutation operator, we use the basic bit mutation, the mutation probability is 0.08, and the mutation position is selected randomly.

Next, we present how to define each operators using HOL4.

The selection operator is defined as follows:

val *Pi* = FST (List.nth (QuickresultList, hd Random.rangelist(0,16) (1,Random.newgen())))

QuickresultList preserves the chromosome and its fitness value according to fitness value from low to high. hd Random.rangelist(0,16) (1,Random.newgen()) indicates the selected chromosome subscript with equal probability. FST deletes the selected fitness value of the chromosome and returns the selected chromosome to the chromosome *Pi*◦

The crossover operator is defined as follows:

fun TWOIPOINT_CROSSOVER *l* (*p*,*q*) = (CROSSOVER *l p q*, CROSSOVER *l q p*)

TWOIPOINT_CROSSOVER is the formalized crossover operator, CROSSOVER is the formalized crossover operation. With the two-point crossover, TWOIPOINT_CROSSOVER *l* (*p,q*) will transfer a cross-term *l* to two crossover points.

The mutation operator is defined as follows:

fun BMUTATION (*pih*::*pil*) *point* = List.take ((*pih*::*pil*),*point*-1) @ ((Random.rangelist(1,11)(1,Random.newgen()) @ List.drop ((*pih*::*pil*),*point*)))

BMUTATION represents the mutation operator, *pih*::*pil* is the chromosomes to be mutated, *point* indicates the mutation point randomly produced. List.take ((*pih*::*pil*), *point*-1) obtains the genes before the mutation point, Random.rangelist(1,11)(1,Random.newgen()) can select the allele gene by equal probability at the mutation position, List.drop ((*pih*::*pil*),*point*) obtains the genes after the mutation point, @ is used to connect the gene segments obtained by the three functions after the mutation.

With the genetic algorithm, we can obtain the optimization collision free path. In order to ensure that the genetic algorithm can find the final path and meet the collision free conditions, we need verify the final path. In this paper, the array of coordinate points of the final path represented by the list. If one of the elements in the list is equal to the coordinate of the obstacle, the final path does not meet the property of collision free.

The verification result is shown as follow:

val *BP* = INTER ([(1,2),(2,4),(3,6),(4,7),(5,8),(6,8),(7,9),(8,9), (9,9),(10,10)],
  [(5,7),(7,8),(8,8),(9,8)]);
  > val *BP* = []: int list

Among them, INNER refers to the formal description of the property of collision free is shown below:

val BP = INTER (*PATH, O*);

It is used to determine whether the two lists have the same elements. If there is no such elements, it outputs the empty list; otherwise, it generates the new list of the same elements. *PATH* is the list of the final robot path generated by the genetic algorithm, *PATH*=[(*x1*,*y1*),…,(*x10*,*y10*)], (*xi*,*yi*)(1 ≤ *i* ≤ 10) indicates the coordinates of the final path. *O* is the list of the Coordinates of the obstacles,*O*=[(*xO1*,*yO1*),…(*xOm*,*yOm*)], (*xOi*,*yOi*)(1 ≤ *i* ≤ *m*) indicates the Coordinates of the obstacles, and *m* represents the number of the obstacles.

The *BP* is final result. It is an empty list, illustrating the optimal or near optimal path generated by the genetic algorithm has no common elements with the obstacles. Thus, the final path meets the collision free condition.

## Conclusion

In this paper, we formalized crossover operations with higher-order logic based on HOL4 that is easy to be deployed with its user-friendly programing environment. We implemented our technique to solve a path planning problem using a genetic algorithm with our formalized crossover operations, and the results show the effectiveness of our technique.

There are two directions for the future work. First, it is interesting to extend formalized crossover operations to other applications such as energy optimization for embedded systems (Wang et al., 2011) and non-volatile memory (Chen et al., 2016; Long et al., 2016; Wang et al., 2016; Liu et al., 2017), and construct a crossover operator library in HOL4. Moreover, we can further formalize genetic algorithms using formalized crossover operators. Based on this, the formalized genetic algorithm can be used to create the general tactics in HOL4, thus improving the automation level of the interactive theorem proving system.

## Author contributions

JZ: Substantial contributions to the conception and design of the work, drafting the work, final approval of the version to be published. MK: Substantial contributions to draft the work the acquisition, analysis, and interpretation of data for the work. XL: Revising the work critically for important intellectual content. GL: In ensuring that questions related to the accuracy of the work are appropriately investigated and resolved.

### Conflict of interest statement

The authors declare that the research was conducted in the absence of any commercial or financial relationships that could be construed as a potential conflict of interest.
